# Identifying entrepreneurial discovery processes with weak and strong technology signals: a text mining approach

**DOI:** 10.12688/openreseurope.14499.2

**Published:** 2022-11-01

**Authors:** Levan Bzhalava, Jari Kaivo-oja, Sohaib S. Hassan, Wolfgang Dieter Gerstlberger

**Affiliations:** 1 Finland Futures Research Centre, Turku School of Economics, University of Turku, Turku, 20500, Finland; 2 Department of Business Administration, TalTech School of Business and Governance, Akadeemia tee 3, Tallinn, 12611, Estonia; 3 Kazimieras Simonavičius University, Vilnius, 02189, Lithuania; 4 SME Management Graduate School, University of Siegen, Siegen, 57072, Germany

**Keywords:** Smart Specialization Strategy (S3), Entrepreneurial Discovery Process (EDP), technology foresight, weak signals, innovations, patents, text mining, topic modelling, word2vec.

## Abstract

This study aims to propose methods for identifying entrepreneurial discovery processes with weak/strong signals of technological changes and incorporating technology foresight in the design and planning of the Smart Specialization Strategy (S3). For this purpose, we first analyse patent abstracts from 2000 to 2009, obtained from the European Patent Office and use a keyword-based text mining approach to collect weak and strong technology signals; the word2vec algorithm is also employed to group weak signal keywords. We then utilize Correlation Explanation (CorEx) topic modelling to link technology weak/strong signals to invention activities for the period 2010-2018 and use the ANOVA statistical method to examine the relationship between technology weak/strong signals and patent values. The results suggest that patents related to weak rather than strong signals are more likely to be high-impact innovations and to serve as a basis for future technological developments. Furthermore, we use latent Dirichlet allocation (LDA) topic modelling to analyse patent activities related to weak/strong technology signals and compute regional topic weights. Finally, we present implications of the research.

## Plain language summary

The European Union (EU) has introduced Smart Specialization Strategy (S3) to increase the innovation and competitive potential of its regions by encouraging them to specialize in limited key economic areas. However, as the quantity of information is growing rapidly in today’s digital economy, regional policy-makers lack efficient and viable tools to interpret external information, knowledge and map promising areas for smart specialization. To address this issue, we used text mining methods to identify entrepreneurial discovery processes with weak/strong signals of technological changes and incorporate technology foresight in the design and planning of S3. For this purpose, we first analysed patent abstracts from 2000 to 2009 provided by European Patent Office and used a keyword-based text mining approach to collect technology weak and strong signals. We then linked technology weak/strong signals to invention activities for the period 2010–2018 and examine the relationship between technology weak/strong signals and patent values. The results suggest that patents related to weak rather than strong signals are more likely to be high-impact innovations and to serve as a basis for future technological developments. Furthermore, we used topic modelling to analyse patent activities related to weak/strong technology signals and compute regional topic weights.

## Introduction

The European Union (EU) has introduced the Smart Specialization Strategy (S3) to promote sustainable and inclusive economic growth in its regions by inducing them to discover and develop economic areas in which they can have comparative and competitive advantage. S3 is a place-based and bottom-up innovation policy which allows the EU regions to tap into their endogenous potential ( Coffano & Foray, 2014; Foray *et al.*, 2009). As knowledge and competencies are dispersed and divided locally, “entrepreneurs in the broadest sense (innovative firms, research leaders in higher education institutions, independent inventors and innovators) are in the best position to discover the domains of R&D and innovation in which a region is likely to excel given its existing capabilities and productive assets” ( Foray & Goenaga, 2013). For this reason, S3 relies on an entrepreneurial discovery process (EDP) to map promising areas for investment and specialization ( Coffano & Foray, 2014). The role of regional governments in S3 is to identify potential entrepreneurial discovery projects and to develop critical mass in these strategic priority areas in order to facilitate micro-level discovery and experimentation processes ( Foray *et al.*, 2011), but policymakers lack appropriate tools and methods to identify and assess promising EDPs. 

Previous research studies the distribution of knowledge claims by regional patents and explores co-occurrence of technology classes to identify industry diversification and specialization opportunities across EU regions ( Balland *et al.*, 2019; Montresor & Quatraro, 2020). Another strand of literature utilizes an unsupervised text mining approach to explore latent topics and to map innovation ecosystems based on startup activities and scientific publications ( Bzhalava *et al.*, 2018; Moilanen *et al.*, 2021). Although the prior research provides valuable insights for understanding specialization patterns across regions, little is known about how to identify and assess EDPs based on technology weak signals. Identifying and interpreting weak signals of impending technological changes are an important part of creating smart specialization strategies ( Paliokaitė *et al.*, 2015; Paliokaitė *et al.*, 2016). Weak signals approach refers to information about past or current developments that can be used to capture uncertain futures and anticipate technological changes ( Kaivo-oja, 2012; Kaivo-oja & Lauraeus, 2018). Weak signals are defined “as seemingly random or disconnected pieces of information that at first appear to be background noise, but which can be recognized as part of a larger pattern when viewed through a different frame or by connecting it with other pieces of information” ( Schoemaker *et al.*, 2013). In other words, they are the early signs for future disruptions and discontinuities, and being able to monitor and analyse weak signals can help decision-makers substantially in predicting impeding technological and business changes ( Kaivo-oja, 2012; Kaivo-oja & Lauraeus, 2018). Hence, incorporating weak signal technology in studying and evaluating entrepreneurial discovery processes can help regional governments to map promising areas of future specialization across territories and to design European S3 ( Paliokaitė *et al.*, 2015; Paliokaitė *et al.*, 2016). Although, prior studies propose automatized tools to detect weak signals of technological changes ( Thorleuchter & Van den Poel, 2013; Thorleuchter *et al.*, 2014; Yoon, 2012), we lack understanding of how to detect entrepreneurial discovery processes with weak/strong signals in an automotive way and how to incorporate technology foresight in the design and planning of European S3. We address this research gap by first collecting weak/strong signals from European patent claim database and then utilize CorEx topic modelling to identify invention with weak and strong signals, as well as employ the ANOVA statistical method to examine whether patent values can be assessed by the weak and strong signals of technological development. We also run latent Dirichlet allocation (LDA) topic modelling to analyse patent activities related to weak/strong technology signals and compute regional topic weights.

## Conceptual framework

By adopting S3, EU aims to avoid duplication and fragmentation of its resources and to increase effectiveness of its research and innovation activities in the face of increased global competition ( Foray *et al.*, 2011; McCann & Ortega-Argilés, 2016). In contrast to traditional industrial and innovation policy in which a decision-making process was mainly centralized and top-down, S3 is a bottom-up collective reflection process in which local actors from industry and academia discover technology and market opportunities and identify promising areas for specialization ( Coffano & Foray, 2014). As knowledge are fragmented and distributed among local actors and setting innovation-policy priorities involve many uncertainties, policymakers usually lack sufficient information to identify future growth opportunities. For this reason, traditional industrial policy and its top-down approach in setting innovation priorities often failed to promote local economic development ( Barca *et al.*, 2012; McCann & Ortega-Argilés, 2016). In particular, “the evidence from numerous development policy examples worldwide demonstrates that regions have made many mistakes in terms of their policy choices, and often this was because policies were chosen on the basis of criteria which were not appropriate or relevant for the local context” ( McCann & Ortega-Argilés, 2016). As old industrial policy relies ‘one-size-fits-all’ strategy solutions and advocates the replication of successful innovation policies applied in very different contexts, it was ineffective to stimulate endogenous economic potential across regions and often caused underdevelopment ( Barca *et al.*, 2012; McCann & Ortega-Argilés, 2016). Moreover, traditional innovation and development policies mainly focus on securing continuity of technology and industrial structure, and for this reason, it placed great emphasis on interests of large established firms in setting innovation priorities ( Asheim, 2019). As incumbent firms mainly rely on the dominant design in their production system and concentrate on generating incremental innovations through exploiting mature technologies and existing knowledge ( Lindholm-Dahlstrand *et al.*, 2019), the traditional industrial policy constrains local innovation activities ( Asheim, 2019; Isaksen & Trippl, 2016). In contrast, S3 focuses on altering and renewing of technology and industrial structures across territories and relies on EDP in identifying future growth opportunities ( Foray, 2015). EDP involves a wide range of local agents from business and academia to discover and produce information about new activities of future specialization, whereas ‘the regional government assesses the activities’ potential and empowers those actors most capable of realizing that potential’ ( Virkkala & Mariussen, 2018). Hence, entrepreneurs (in the broadest sense) explore new market opportunities and bring into existence novel ideas and technologies as well as reshape markets and value networks across industries ( Bzhalava *et al.*, 2017; Bzhalava *et al.*, 2022; Lindholm-Dahlstrand *et al.*, 2019; Schumpeter, 1934). This process is termed as “creative destruction” in the classic innovation literature ( Schumpeter, 1934). In “creative destruction” process entrepreneurial firms bring new technologies to the market and out-compete established companies by cannibalizing their market profit (which based on existing technologies) and making the value of their accumulated knowledge obsolete. This, in turn, leads shifting profit pools from incumbents to new firms, as well as rearranging industry structures and replacing established businesses ( Lindholm-Dahlstrand *et al.*, 2019). Hence, by relying on EDP, European S3 aims to implement structural economic changes across regions through identifying new domains of future opportunities in which they can have competitive advantages and concentrating resources on those key limited areas ( Foray, 2015).

Literature in evolutionary geography of innovation shows that there are significant differences across regions in terms of innovation activities ( Balland & Boschma, 2021; Boschma, 2017; Feldman & Kogler, 2010; Rigby *et al.*, 2022). The ‘place-based’ character explains regional differences regarding innovation patterns and intensity with reference to the varying economic, technological, cultural and historic contexts of different regions ( Boschma, 2017). In other words, varies regional contexts influence private, public and civil society stakeholder’s behaviour differently and these process influences the whole range of innovation types, from social and organisational innovations to process and product/service innovations. In this context, EDP is crucial for discovering innovation activities in which regions can have competitive and comparative advantages and for defining regional smart specialisation strategies ( Marinelli & Perianez-Forte, 2017). This process ‘is about prioritising investments based on an inclusive and evidence-based process driven by stakeholders’ engagement and attention to market dynamics’ ( European Commission, 2022). Although the financial and organisational necessity of such a systematic exploration process for a successful smart specialisation process seems to obvious, the reviewed literature does not provide clearly structured paths for conducting an EDP. Besides the question regarding the necessary content of an EDP (e.g. in relation to financial, technological, and organisational aspects), there is also a lack of methodological clarity. EDP methods that researchers have applied in the last years range from qualitative expert interviews or focus groups, quantitative secondary data analyses or (cross-sectional) surveys to more complex mixed-method foresight studies with different waves of data collection/analysis ( Gheorghiu *et al.*, 2016; Perianez-Forte *et al.*, 2021). For example, Balland *et al.* (2019) uses co-occurrence analysis and explores patent technology classes in regional patent databases to examine regional knowledge bases and to identify technological upgrading (diversification) opportunities. Similarly, Drivas (2020) studies trademark business classifications to detect future growth opportunities across European regions ( Drivas, 2020). In contrast, Moilanen *et al.* (2021) employ an unsupervised text mining method to detect latent topics and thematic networks in scientific literature. Other strand of literature utilizes network analysis to examine startup company sub-industry tags and business descriptions to explore the structure of entrepreneurial ecosystems and to examine where entrepreneurs drive their businesses across different sectors and territories and to detect specialization patterns ( Basole *et al.*, 2018; Losurdo *et al.*, 2019). Furthermore, Papagiannidis *et al.* (2018) use an unsupervised text mining approach to discover latent topics in websites of firms and visually identify areas of intense business activities across various places. Similarly, other scholars explore web pages of firms to map innovation ecosystems ( Beaudry *et al.*, 2016; Kinne & Axenbeck, 2020). In particular, as web pages of companies are often used to publish information about innovative products/services, previous research uses web and text mining methods to survey business websites and to detect innovation activities across territories ( Beaudry *et al.*, 2016; Kinne & Axenbeck, 2020). 

To understand future technology and market landscape and to identify promising EDPs, foresight can be significantly helpful as an instrument to study the potential ways in which the future technology and market landscape can unfold. Specifically, foresight is defined ‘as a process which involves systematic inquiry into longer-term futures, including emerging and novel issues, which in turn enables present decision-making and action’ ( Minkkinen *et al.*, 2019). In the process of strategic planning with foresight, a well-known approach is to identify and analyze weak signals which are the early signs of future disruptions and discontinuities ( Thorleuchter & Van den Poel, 2015). A weak signal is an indicator of a potentially emerging issue, that may become significant in the future ( Holopainen & Toivonen, 2012). Weak signals or peripheral visions are often identified as a part of horizon scanning (or environmental scanning) that supplements trend analysis and can be used as a foundation for defining wild cards ( Day & Schoemaker, 2004; Kaivo-oja, 2012; Mendonça *et al.*, 2004). We can note that weak signals can become strong signals or stay weak signals. In the first case, weak signals are likely to transform into high-impact innovations. Both demand and supply factors can push weak signals to become strong signals. In the second case, weak signals do not transform into high-impact innovations, and there are no demand and supply factors to make weak signals become strong signals. In social or business communities, there are change agents who typically are pioneers, who identify weak signals and adopt them first. Latecomers follow the pioneering change agents, and weak signals change to strong signals and to so-called micro trends. Microtrends can be adopted in the established socio-technical regimes (macro trends at the macro level) and later even in the global economy (global trends of the global landscape) ( Geels, 2011; Mylan *et al.*, 2015).

To collect technology and business related weak signals, prior studies propose text mining methods ( Kim & Lee, 2017; Thorleuchter & Van den Poel, 2013; Thorleuchter & Van den Poel, 2015; Yoon, 2012). In particular, Yoon (2012) develop a keyword-based weak signal detection approach to assess the strength of the terms for each topic by measuring keyword frequency (visibility) and the degree of diffusion-based on document frequency, as well as calculates time-weighted increasing rates of keywords based on their degree of visibility/diffusion and computes average term (document) frequency to identify weak and strong signal related terms. Similarly, Kim and Lee (2017) propose the novelty-focused weak signal detection approach by first applying text mining to extract signals from documents and then employing a local outlier factor to study the rarity and paradigm unrelatedness of weak signals. Although prior research develops big data and text mining tools to identify weak signals of impeding technological and business changes, we lack an understanding of how to incorporate technology foresight in the design and planning of European S3. To address this issue, the research aims to identify and assess EDPs (e.g. invention activities) with technology weak and strong signals. It is important to apply Big Data analytics to understand EDPs. Specifically, we incorporate weak signal technology analysis in studying and evaluating entrepreneurial discovery processes that can help regional governments to map promising areas of future specialization across territories and to design European S3.

## Data and methodology

In this research, we used patent claim data from the European Patent Office (EPO). EPO provides access all European Patent (EP) publications from 1978 until the end of January 2019. EP publications are in XML, PDF and TIFF formats and this makes it difficult to extract only publication text data. To address this issue, EPO created EP full-text data for text analytics and provides free and easy access to EP publication text data, which includes information about the publication authority, number, publication kind, date, the language of text component, text type (i.e. title, abstract, description of the invention, a set of claims) as well as the publication abstract and the full-text description of the invention. As a patent abstract text includes essential information about an invention and its technical details ( Lee *et al.*, 2019), we used patent claim abstract data in our research and extracted only abstracts with English language from EP full-text data (for further details, see *Underlying data*). In the study, we aim to identify invention activities associated with weak/strong signals of technological changes and to incorporate technology foresight in the design and planning of S3. For this purpose, in line with previous research, we first collected weak/strong technology signals from patents abstracts during ten years period from 2000 to 2009 ( Kwon *et al.*, 2018; Yoon, 2012), and then we linked weak/strong technology signals with invention activities during 2010-2018 time period. The chosen period 2010-2018 regarding invention activities reflects the most relevant smart specialisation policy documents and activities of the European Commission, which started in around 2010.

To identify regional dimension of patent abstracts (for 2010–2018 time period) and to measure their quality, we combined EP data with the OECD-REGPAT and OECD Patent Quality Indicators databases by using the patent publication number. The OECD-REGPAT database January 2020 allows to connect patent data with regional dimension by using the addresses of the applicants and inventors. The OECD Patent Quality Indicators database July 2020 provides information about indicators of patent technological and economic values (e.g. number of citations a patent received up to five years after publication, and if a patent belongs to the top 1% highly cited patents up to five years after publication).

### Identifying weak signals with keyword-based text mining

We clean and pre-process patent abstracts, which involved transforming all textual content into lower case keywords and deleting numbers, non-English and special characters, as well as removing punctuations and stop-words. Moreover, keywords are lemmatized, which refers to applying vocabulary and morphological analysis and transforming keywords into their root forms. We then extracted only noun and adjective keywords as they represent technology concepts.

Terms associated with weak signal topics usually have a low absolute occurrence frequency and a high time-weighted increasing rates. On the contrary, terms related to strong signal topics are likely to have a high absolute occurrence frequency and a high time-weighted increasing rates. In line with Yoon (2012), we study the occurrence of keywords and uses a time-weighted method to put recent appearances of keywords more important than past appearances. Specifically, we calculated their Degree of Visibility (DoV) based on the frequency of their occurrence and Degree of Diffusion (DoD) based on their appearance in number of patent abstracts ( Yoon, 2012). Mathematically, DoV and DoD are expressed in the following way:

$$DoV_{ij}=\left(\frac{TF_{ij}}{NN_{j}}\right)\times \{1-tw\;\times (n-j)\}\;(1)$$

$$DoD_{ij}=\left(\frac{DF_{ij}}{NN_{j}}\right)\times \{1-tw\;\times (n-j)\}\;(2)$$

where *TF _ij_* refers to the total occurrence frequency of a term *i* in period *j*, and *DF _ij_* denotes to the document frequency of term *i* in period *j*. *NN _j_* stands for the total number of patent abstracts in period *j*, whereas n is the number of periods. Furthermore, to put more weight on recent appearances of keywords and to give recent occurrences of keywords more importance than past occurrences, Yoon (2012) introduce tw (a time-weight) which is defined as 0.5. Moreover, the geometric mean is calculated based on DoV and DoD values to explore the increasing rates of keyword occurrences. Terms associated with weak signal topics usually have a low absolute occurrence frequency and a high time-weighted increasing rates. On the contrary, terms related to strong signal topics are likely to have a high absolute occurrence frequency and a high time-weighted increasing rates. In line with Yoon (2012), we extract keywords that are in the top 30% in terms of growth rate. Finally, we consider keywords that have less than average absolute yearly term (document) frequency as weak signals, and those with more than average absolute yearly term (document) frequency as strong signals.

By analyzing patent abstract data for the period 2000-2009, we identified weak and strong signal keywords related to hospitality/travel, education, telecommunication, healthcare, media and entertainment, environment, transportation and construction industries. Specifically, the following weak signal keywords were identified for the hospitality/travel industry: cooking, accommodation, wine, leisure. For the education industry – education; for the telecommunication industry – cell phone; for the healthcare industry – care, health, vaccine, symptom, anticancer. For the media and entertainment industry – advertisement, media, gaming; for the environment technologies/industries – planet, biosensor, biomass, greenhouse, sustainability; for the transportation industry – motorcycle, flight; for the construction industry – cement, construct. Afterwards, we utilised the word2vec algorithm to group weak signal keywords ( Mikolov *et al.*, 2013). In particular, we first kept only weak signal keywords in patent abstracts and removed other words. We then utilised word2vec to train text corpus and extract terms that have high correlation (0.70–0.99 range) with the industry keywords; the word2vec algorithm uses a neural network approach to create word embeddings by learning word associations (co-occurrence) from text and locating words with similar meaning close to one another in the vector space ( Mikolov *et al.*, 2013). Strong signal keywords were also classified into telecommunication, healthcare, media and entertainment, environment and transportation sectors. Weak and strong signal keywords and their total frequencies, as well as their Degree of Visibility (DoV) and Degree of Diffusion (DoD) values are presented in ‘Extended Data’ (for further details, see *Underlying data*).

### Linking weak/strong signals to patent abstracts

After collecting weak and strong signal related keywords, we linked them with EU patent abstract data for the period 2010–2018. For this purpose, we first cleaned and pre-processed textual context of patent abstracts and then utilised Correlation Explanation (CorEx) topic modelling, which is a semi-supervised text mining approach that allows the defining of “anchor words” and guides learning topics in the direction of those anchor words ( Gallagher *et al.*, 2017). In other words, we use weak and strong signal keywords as anchor words and use CorEx topic modelling to identify related invention activities across EU regions for the period 2010-2018. In total, we identify 366,392 patents associated with weak and strong signals. Moreover, we apply latent Dirichlet allocation (LDA) to discover hidden topics in those patent abstracts. LDA is an unsupervised machine learning method which automatically creates cluster terms that have a higher possibility of showing up together and reveals latent topics in a collection of documents ( Blei *et al.*, 2003). To decide the number of topics to search in patent abstracts and to measure the quality of the topics discovered, we tested a range of topic numbers from 2 to 50 and calculated coherence scores (ibid). Finally, we selected topics with the highest coherence score and ran LDA with 7 topics.

Furthermore, we employed a one-way ANOVA modelling to study the relationship between weak/strong signals and patent values. Patent quality is usually measured by the number of forward citations it receives ( Briggs & Buehler, 2018; Hall *et al.*, 2005). A patent is considered as breakthrough if it receives a disproportionately large number of forward citations and has a considerably large impact on subsequent technological progress ( Arts & Veugelers, 2015; Squicciarini *et al.*, 2013). In line of prior research, we measured a patent quality by (1) number of citations it receives up to five years after publication and also (2) if it belongs to the top 1% highly cited patents up to five years after publication. The first one is a continuous variable, whereas the second one is a binary variable - 1 if a patent is classified as a breakthrough and 0 otherwise ( Squicciarini *et al.*, 2013).

## Results

The results show that major invention activities related to weak signals of technological changes are concentrated in sustainability, anticancer, symptom, vaccine and greenhouse areas, whereas patent activities associated with strong signal keywords are concentrated in mobile technologies, healthcare and aircraft/vehicle areas (see Table 1– Table 2). Moreover, we also examined whether patents associated with weak or strong signals have more technological and economic values in terms of number of citations a patent received up to 5 years after publication, and if a patent belongs to the top 1% highly cited patents up to 5 years after publication. By employing the one-way ANOVA modelling approach, the results suggest that patents related to technology weak rather than strong signals are more likely to be high-impact innovations and to serve as a basis for future technological developments (see Table 3– Table 6). Technology foresight can provide vital input in the design and planning of European S3 in terms of providing information about emerging technologies, discontinuities and potential future threats and opportunities.

**Table 1a.** Weak signal keywords and related invention activities.

Weak signal keywords	Number of related patents
**Accommodation**, shuttering, camping	1098
**Advertisement**, commercial, advertise, webpage, publishing, prompting, friend, online, sms, choose, notify, renew, advertising	1606
**Anticancer**, cytotoxicity, metastasis, somatostatin, hepatitis, radionuclide, ceramide, pyrimidine, antitumor, quinazoline, apoptosis, macrophage, liposome, histocompatibility, rodent, immunology, carcinoma, alkanoyl, neovascularization, autoimmune, tautomer, arginine, choline, monocyte, mammalian, malignancy, reproducible, chemotherapy, death, fibrosis, administering, integrin, dystrophy, fertility, immunosuppression, mitochondrial, methionine, interferon, bioactivity, schizophrenia, cytoplasm, suffering, depolymerization, fever, arthritis, pancreas, mortality, mucosal, solvate, potency, prophylaxis, proliferation, hydrate, angiogenesis, fertilization, pathology, mimetic, therapeutic, sepsis, formyl, methoxy, lymphocyte, chimeric, phospholipase, segregating, phospholipid, piperidine, liver, tcell, hydrolase, diabete, tuberculosis, carboxy, brightener, thrombosis, prodrug, kidney, toxin, transplant, sclerosis, homology, osteoarthritis, fibroblast, marrow, cyclohexyl, defective, colon, tyrosine, atherosclerosis, metabolite, tumour, thiazole, polyphosphate, mononuclear, bowel, amyloid, ventricular, germ	10273
**Biomass**, gasification, refinery, bioreactor, fermentation, ethanol, pepper, grape, broth, nitrate, fossil, greenhouse, herb, silage, maize, wine	1851
**Biosensor**, analyte, biomolecule	1222
**Cellphone**	27
**Cement**, slump, clinker, rheology, copolymerisation, translucency, consolidation, alkalinity	1132
**Cooking**, cooker, stove, digester, utensil, blowoff, supercapacitor, recipe, pastry	1590
**Construct**, modularity	520
**Education**, inability, aviation, consultation, extrude, sharpener, online, intervene, avionic, demarcation, disaster, society, searching, ventricular	750

**Table 1b.** Weak signal keywords and related invention activities.

Weak signal keywords	Number of related patents
**Flight**, waypoint, airframe, airport, spanwise, refueling, avionic, tanker, craft	1969
**Gaming**, multiplayer, redemption, proceeding, outcome, online, distraction, voucher, friend, adoption, ranking	1039
**Greenhouse**, silage, stalk, harvest, borne, pepper, cultivation, vegetation, grape, fungi, farm, biomass, shredder, distillate, crop	2016
**Health**, Care, consultation, hospital, diet, linoleic, polyphenol	1810
**Leisure**, entertainment, online, aviation, playing	116
**Media**, renderer, playlist	573
**Motorcycle**, fender, windscreen, handgrip, headlight	920
**Planet**, gearing, sun, bogie	699
**Sustainability**, poise, hydrogenating, anisotropy, butene, cyclohexyl, cmin, overgrowth, carbonylation, borohydride, prescribed, durometer, sharpener, methoxy, govern, society, novolac, diblock, thiazole, shelving, displaceable, carboxymethyl, cumbersome combust, ventricular, electrovalve, brightener, screwing, red, recyclability, extrude, hydroxylamine, ignitability, discs, microfabrication, flammability, potting, thioester electrocatalyst, flocculating, protecting, lactide, polyisoprene, alkanoyl, envisage, bioactivity, copolymerisation, sparking, piperidine, varnish, stirring, ceramide, nitrate, saliency, counteracting, alkylphenol, volatilization, rodent, accretion, flaking, praseodymium choline, depolymerization, evaporate, oscillate, monoglyceride, citric, linoleic, diene, terpene onpress, brightening, granulometry, dimethyl, interpolate, coatability, gadolinium, sulfonate, polyamine, quinazoline, dyestuff, nanocomposite deformability alkalinity, bedplate vaporizing, polyphenol, chlorination, tautomer, pepper microdroplet, rutile, tetrachloride, calcination, sputtering, polyanion, cranking, thermoformable, pyrimidine, lipid, phospholipid, tampon, maize ferric, acetylene inject diamine dimerization, carboxy, atomizer, cytoplasm, arginine, avionic, radiant, phenylene, phospholipase, formability, aluminosilicate, somatostatin rosin amlodipine turbomachine, dipeptide, polyphosphate methionine, formyl	47525
**Symptom**, bowel, amelioration, sepsis, sclerosis, alleviation, schizophrenia, osteoarthritis, prophylaxis, administering, atherosclerosis, syndrome, arthritis, hyperactivity, dementia, antiobesity, alzheimer, inflammation, piperidine, mood, suffering, obesity, mellitus, deficit, analgesic, potency, pathology, abuse, hypertension, fever, endocrine, athlete, overgrowth, hyperglycemia, allergy, pain, amlodipine	3364
**Vaccine**, tuberculosis, vaccination, antigen, immunology, chicken, allergy, pathogen, hepatitis, chimeric, mammalian, tcell, histocompatibility, fertilization, replicating, rodent, epitope, hcv, therapeutic, lymphocyte, immunosuppression, interferon, mucosal, carcinoma, neovascularization, fertility, mimic, antitumor, depolymerization, pathology, cytoplasm, monocyte, inactivation, toxin	3042
**Wine**, grape, pepper, cappuccino, brewing, herb, pastry, coffee, fermentation, lactide, alkylphenol, apartment, broth, bedplate, drink, flour, biomass	1509

**Table 2.** Strong signal keywords and related invention activities.

Strong signal keywords	Number of related patents
Aircraft, turbine, vehicle, chassis	11955
Broadcast, multimedia, transmit, receptor, display, radio	10466
Cancer, peptide, tumor, medicament, antibody, pharmaceutical, nucleic, disease, molecule, therapy, precursor, amino, irradiation, drug, patient, virus, treatment, cell, membrane	51710
Lighting, dust, washing, tissue, luminance, security, encoding, tire, protein, storage, sensor	9907
Mobile	192409
Wind, emitting, plant	5294

**Table 3.** Forward citation (five year) and strong/weak signals, ANOVA analysis.

	sum_sq	DF	F	PR(>F)
C(strong_weak_signals)	2.083034e+03	1.0	46.616547	8.646175e-12
Residual	1.637193e+07	366390.0	NaN	NaN

**Table 4.** Forward citation (five year), average.

	forward citation (5 year)		
Weak signal related patents	1.665
Strong signal related patents	1.486

**Table 5.** Breakthrough inventions (1% most citied patents) and strong/weak signals, ANOVA analysis.

	sum_sq	DF	F	PR(>F)
C(strong_weak_signals)	0.165795	1.0	20.504968	0.000006
Residual	2962.482775	366390.0	NaN	NaN

**Table 6.** Breakthrough inventions (1% most citied patents), average.

	Breakthrough inventions		
Weak signal related patents	0.009
Strong signal related patents	0.007

To study invention activities across regions that are associated with weak and strong signals of technological changes, we used LDA topic modelling to discover hidden themes in patent abstracts. We focused on the most frequent words that are important to interpret and distinguish topics. Looking at the results of LDA topic modelling, Table 7 shows that major invention activities associated with water technologies (Topic 0), cell domain/embodiment (Topic 1), image display (Topic 2), protein acid (Topic 3), data systems (Topic 4), sensor-based intelligence systems (Topic 5) and aircraft/vehicle power systems (Topic 6). Moreover, we investigated the prevalence of each topic by calculating region topic proportions. By assuming that documents are a probability distribution of topics and topics are a probability distribution of words, LDA calculates probability distributions that a document (in our case a patent abstract) is associated with multiple topics. Hence, LDA allows us to present documents with corresponding topic probabilities and to show how different topics are distributed over patent abstracts as well as to calculate regional patent weights. After LDA analysis, we obtained probability vectors over topics for each patent abstract. We sum topic probabilities of patent abstracts in each EU regions to study which European regions in which areas/topics contribute invention activities.

**Table 7.** The top 10 ranked words of selected weak/strong signal related topics in patent activities.

Topic 0		Topic 2		Topic 4		Topic 6	
*Word*	*probability*	*word*	*probability*	*Word*	*probability*	*word*	*probability*
washing	0.031	portion	0.021	display	0.030	vehicle	0.071
method	0.021	display	0.020	information	0.021	tire	0.035
water	0.017	direction	0.016	Data	0.017	turbine	0.032
plant	0.016	surface	0.016	device	0.017	control	0.023
invention	0.015	first	0.015	First	0.015	wind	0.019
dust	0.015	body	0.013	method	0.013	system	0.018
present	0.011	side	0.011	security	0.012	aircraft	0.018
rubber	0.011	image	0.011	One	0.012	power	0.016
step	0.008	second	0.011	system	0.010	air	0.012
tub	0.007	position	0.011	Unit	0.010	unit	0.071
Topic 1		Topic 3		Topic 5			
*word*	*probability*	*word*	*probability*	*word*	*probability*		
cell	0.038	invention	0.031	signal	0.032		
base	0.031	protein	0.021	Light	0.021		
bead	0.019	present	0.017	Value	0.020		
channel	0.017	tissue	0.017	First	0.016		
shoulder	0.015	comprising	0.015	Unit	0.015		
block	0.014	relates	0.013	second	0.013		
domain	0.011	methods	0.012	sensor	0.012		
embodiments	0.010	acid	0.012	based	0.012		
include	0.010	said	0.010	detection	0.010		
may	0.009	proteins	0.010	Time	0.009		

The top EU regions with the highest probability distributions of patent abstracts in the selected topics are present in Table 8. These probability distributions indicate how the different EU regions contribute to each individual topic, demonstrating how they are concentrating their invention activities on specific topics, reflecting R&D priorities. For example, the results show that Paris has the highest regional topic weights in water technologies (Topic 0) and protein acid (Topic 3), whereas München specialize in aircraft/vehicle power systems (Topic 6). Moreover, Hauts-de-Seine is strongly presented in the following invention activities such as cell domain/embodiment (Topic 1), image display (Topic 2), data systems (Topic 4) and sensor-based intelligence systems (Topic 5). By employing interdisciplinary elements of economic innovation, foresight and big data fields, the research linked technology foresight and regional innovation activities, as well as explored possibilities of identifying and assessing entrepreneurial discovery processes with weak and strong signals of technological changes. 

**Table 8.** Regional topic weights in patent activities.

Region	Topic 0	Topic 1	Topic 2	Topic 3	Topic 4	Topic 5	Topic 6
Hauts-de-Seine	347.5	394.2	254.8	475,1	2894,1	701,1	137,7
Paris	414.4	259.1	147.8	2061,1	1449,3	376,5	132,1
München	346.1	143.1	224.4	688,5	983,5	449,6	467,3
Stockholm country	165.1	222.3	127.7	385,4	1216,4	288,2	168,7
South-East North Brabant	138.3	145.7	148.9	127,7	1002,5	620,3	353,6
Helsinki-Uusimaa	189.2	157.9	138.7	173,9	1312,2	272,2	115,7
Inner London West	217.6	79.6	177.1	344,2	754,5	188,3	200,3
Madrid	259.3	86.5	143.3	638,3	519,8	119,7	124,2

## Discussion and conclusion

This research presents a text mining approach to detect entrepreneurial discovery processes with weak/strong signals and explores the possibilities of incorporating technology foresight in the design and planning of European S3. The empirical study demonstrates how Big Data sets can be analysed in different European regions. Specifically, we analysed patent claim database and extracted weak/strong signals. We then utilised CorEx topic modelling to identify invention activities with weak and strong signals, as well as employ the ANOVA statistical method to examine whether patent values can be assessed by the weak and strong signals of technological development. In the final stage, an unsupervised text mining approach was used to map European patent activities related to weak/strong technology signals and compute regional topic weights. The results reveal in which areas different EU regions contribute and also reflect R&D priorities. The proposed approach can be used to envision future innovation ecosystems and study weak signal related invention activities across territories, as well as to calculate regional topic weights and develop policy road mapping for innovation ecosystem development in the EU.

In addition, this study shows that patents related to weak technology signals rather than strong, are more likely to be high-impact innovations and to serve as a basis for future technological developments, implying that weak signals matter for detecting breakthrough inventions. As the quantity of information is growing rapidly in today’s digital economy, decision-makers often lack appropriate tools and methods to process massive amounts of external information, interpret signals of impeding technological changes and use them in strategic and innovation management. The proposed approach in this study can help policy-makers and companies to widen the ecosystem-foresight lens and to detect future technology threats and opportunities.

The analysis presented in the research can be replicated to examine relationship between weak/strong signals and start-up performance. Specifically, the research can be extended to identify start-up entrepreneurial activities across territories with technology weak and strong signals. As start-up entrepreneurs explore new market opportunities and bring into existence novel business models, they reshape markets and value networks across industries. Therefore, studying entrepreneurial activities with weak signals can help firms and policymakers to anticipate where ‘creative destruction’ will unfold and in which business areas they should expect business discontinuities driven by new technologies and business models.

The most important limitation of this article is its focus on domains where patenting is key. The consequence of this focus is that this study only investigates specific, more technology-based aspects of smart specialization. Although these specific aspects are of high importance in the smart specialization literature ( D’Adda *et al.*, 2019; Natalicchio *et al.*, 2021), they limit the analysis of this study to patentable product innovations. Process innovations, most forms of service innovations and organizational innovations are excluded. This limitation implies, for example, that innovations in fields like tourism, cultural industries or social service are completely or at least mainly outside the scope of this investigation ( Weidenfeld, 2018). Moreover, the chosen methodological approach also limits the geographical scope of this work. More in detail, e.g. peripheral regions which concentrate their smart specialization strategies on rather simple (in terms of applied technology) touristic and/or cultural services are not considered ( Rigby *et al.*, 2022). However, in relation to patentable innovations, the present study goes beyond the state of the art as described in the relevant literature. In particular, this work suggests an innovative as well as robust methodological and data-related extension.

## Ethics and consent

Ethical approval and consent were not required.

## Data Availability

All data underlying the results are available from the European Patent Office (EPO). This project contains the following underlying data: https://www.epo.org/searching-for-patents/data/bulk-data-sets/data.html#tab-1 https://doi.org/10.6084/m9.figshare.19130729.v2 https://figshare.com/articles/figure/Extended_data_docx/19146494
