# Supplementary material for: Identifying entrepreneurial discovery processes with weak and strong technology signals: a text mining approach
Source: Open Res Eur. 2022 Nov 1;2:26. Originally published 2022 Feb 28. [Version 2] doi: 10.12688/openreseurope.14499.2 (PMC10445809; doi:10.12688/openreseurope.14499.2)
Supplement: Peer review history

# Peer review: Reviewer response for version 2

**DOI:** 10.21956/openreseurope.16453.r30367

**Published:** 2022-12-08

**Authors:** Hugo Pinto

**Affiliations:** 1 Faculdade de Economia, Universidade do Algarve, Faro, Portugal; 2 Centro de Estudos Sociais, Universidade de Coimbra, Coimbra, Portugal

The corrections included by the authors in the revision are in line with my suggestions. Good luck with your future research in this topic.

If applicable, is the statistical analysis and its interpretation appropriate?

Yes

Is the study design appropriate and is the work technically sound?

Yes

Is the work clearly and accurately presented and does it engage with the current literature?

Yes

Are the conclusions drawn adequately supported by the results?

Yes

Are sufficient details of methods and analysis provided to allow replication by others?

Partly

Are all the source data and materials underlying the results available?

Yes

Reviewer Expertise:

Regional Economics, Economic Geography, Specialist in Smart Specialisation, Quantitative Studies

I confirm that I have read this submission and believe that I have an appropriate level of expertise to confirm that it is of an acceptable scientific standard.

# Peer review: Reviewer response for version 2

**DOI:** 10.21956/openreseurope.16453.r30366

**Published:** 2022-11-09

**Authors:** Muhammad Ali

**Affiliations:** 1 Friedrich Schiller University, Jena, Germany

Thank you for addressing my comments. I have read the responses and I am satisfied with them. All the best to the authors.

If applicable, is the statistical analysis and its interpretation appropriate?

Yes

Is the study design appropriate and is the work technically sound?

Yes

Is the work clearly and accurately presented and does it engage with the current literature?

Yes

Are the conclusions drawn adequately supported by the results?

Yes

Are sufficient details of methods and analysis provided to allow replication by others?

Partly

Are all the source data and materials underlying the results available?

Yes

Reviewer Expertise:

Economics of innovation, national system of innovation

I confirm that I have read this submission and believe that I have an appropriate level of expertise to confirm that it is of an acceptable scientific standard.

# Peer review: Reviewer response for version 1

**DOI:** 10.21956/openreseurope.15652.r29176

**Published:** 2022-05-19

**Authors:** Hugo Pinto

**Affiliations:** 1 Faculdade de Economia, Universidade do Algarve, Faro, Portugal; 2 Centro de Estudos Sociais, Universidade de Coimbra, Coimbra, Portugal

The article “Identifying entrepreneurial discovery processes with weak and strong technology signals: a text mining approach” is a relevant contribution to the literature on smart specialization. It is centered in the textual analysis of patent abstracts from 2000 to 2009 provided by EPO and uses a mining approach to collect technology weak and strong signals, linking these signals to invention activities for the period 2010–2018.

 The article presents several good ideas. Nevertheless, I recommend some corrections for the consideration of authors.

- First, smart specialization is a process that is far from being centered in technological diversification. It is anchored in the development of domains where a specific region can excel for its structural change. These domains can be technological intensive or not. This standpoint means that the paper only addresses the entrepreneurial discovery processes that are highly connected with domains where patenting is key – usually more technological. Domains where DUI learning modes are dominant are neglected in this way. And they are crucial, especially in peripheral and/or lagging regions. This limitation should be discussed and referenced in the text.
- Secondly, being the text about the EDP, few lines are spent in clarifying the concept or giving evidence of the many (disparate) ways regions have tried to engage to actively direct the EDPs. Sometimes EDPs were directly related with findings using quantitative evidence-based approaches but were also inspired by participatory and qualitative approaches. A small summary of these practices can be presented to justify the pertinence and gap that this specific approach intends to solve.
- Thirdly, S3 is commonly referred to as a place-based strategy. There is room to accommodate a stronger regional focus is this article. In two ways. In a theoretical way, many contributions from the evolutionary geography of innovation, are directly aligned with this approach, focusing patent portfolios, please cf. many recent contributions of Ron Boschma and co-authors and about relatedness. In the empirical way, the results (that are already presented) could emphasize this matter of regional smart specializations and regional diversification in EU.
- Finally, the option concerning the selection of periods is not clear. It deserves more justification.

Revise the word “citied”.

 Consolidate the decimals in the tables, in particular ANOVA.

 Tables referring topics should revised. Instead of “Topic 1, Topic …” insert the full name of the Topics: (Topic 0), cell domain/embodiment (Topic 1), image display (Topic 2), protein acid (Topic 3), data systems (Topic 4), sensor-based intelligence systems (Topic 5) and aircraft/vehicle power systems (Topic 6).

 Good luck!

If applicable, is the statistical analysis and its interpretation appropriate?

Yes

Is the study design appropriate and is the work technically sound?

Yes

Is the work clearly and accurately presented and does it engage with the current literature?

Yes

Are the conclusions drawn adequately supported by the results?

Yes

Are sufficient details of methods and analysis provided to allow replication by others?

Partly

Are all the source data and materials underlying the results available?

Yes

Reviewer Expertise:

Regional Economics, Economic Geography, Specialist in Smart Specialisation, Quantitative Studies

I confirm that I have read this submission and believe that I have an appropriate level of expertise to confirm that it is of an acceptable scientific standard, however I have significant reservations, as outlined above.

# Response

**Published:** 2022-05-30

**Authors:** Levan Bzhalava

Dear Dr. Pinto,

 We would like to thank you for the effort and expertise that you contributed towards reviewing the manuscript. We sincerely appreciate all your comments and suggestions, which are all valuable and very helpful for revising and improving the manuscript.  

 With best wishes,

 Levan Bzhalava

# Response

**Published:** 2022-10-12

**Authors:** Levan Bzhalava

**Affiliations:** Tallinn University of Technology, Estonia

*We would like to thank the reviewer for constructive comments and suggestions, which helped us considerably to improve the manuscript. Below we provide responses to the reviewer's comments:  *

- First, smart specialization is a process that is far from being centered in technological diversification. It is anchored in the development of domains where a specific region can excel for its structural change. These domains can be technological intensive or not. This standpoint means that the paper only addresses the entrepreneurial discovery processes that are highly connected with domains where patenting is key – usually more technological. Domains where DUI learning modes are dominant are neglected in this way. And they are crucial, especially in peripheral and/or lagging regions. This limitation should be discussed and referenced in the text.

*Author Response: W* *e included discussion regarding limitations of the research in the Discussion and Conclusion section. *

- Secondly, being the text about the EDP, few lines are spent in clarifying the concept or giving evidence of the many (disparate) ways regions have tried to engage to actively direct the EDPs. Sometimes EDPs were directly related with findings using quantitative evidence-based approaches but were also inspired by participatory and qualitative approaches. A small summary of these practices can be presented to justify the pertinence and gap that this specific approach intends to solve.
- Thirdly, S3 is commonly referred to as a place-based strategy. There is room to accommodate a stronger regional focus is this article. In two ways. In a theoretical way, many contributions from the evolutionary geography of innovation, are directly aligned with this approach, focusing patent portfolios, please cf. many recent contributions of Ron Boschma and co-authors and about relatedness. In the empirical way, the results (that are already presented) could emphasize this matter of regional smart specializations and regional diversification in EU.

*Author Response:* *W​​​​​​e extended the literature review and provided more in-depth discussion regarding entrepreneurial discovery processes (EDPs) and a place-based innovation strategy as well as presented quantitative and qualitative approaches concerning many different ways regions have tried to engage to actively direct EDPs (please see* *the Conceptual Framework section* *)*

- ​Finally, the option concerning the selection of periods is not clear. It deserves more justification.

*Author Response:* *We included an explanation regarding the selection of periods.*

# Peer review: Reviewer response for version 1

**DOI:** 10.21956/openreseurope.15652.r28962

**Published:** 2022-05-04

**Authors:** Muhammad Ali

**Affiliations:** 1 Friedrich Schiller University, Jena, Germany

I had the pleasure of reading this paper. It is a very nicely done work with a potentially significant impact on policy. Following are my comments:

1. The authors present a novel approach to predicting signals. In order to show that their methodology is better or more sophisticated, the authors should explain how their methodology is different from the existing methodologies.
2. In the methodology section, the authors write that they merge two REGPAT databases by the address of the authors. Since one of the indicators measures the impact of patents after a lag of a few years, I wonder if the addresses in the two databases are kept the same at the point of publication or if they update it later? If the latter is true, then how did the authors account for the cases where authors changed their address?
3. I guess there are typos in Equations 1 and 2. “NNij” should be “NNj”.
4. It is not clear to me how the term “tw” is used. If it is kept fixed at 0.05 then what purpose does it serve? My guess is that the value of “tw” is conditional on the time elapsed since publication. Whatever the case might be, it needs better clarification.
5. For those who are new to text mining, it would be helpful to know how the authors dealt with synonyms. For instance, “playing”, “lighting”, etc. can have several meanings when used as keywords. How did the authors ensure that they selected the correct patents?
6. How can weak signals be more likely to transform into high-impact innovations? Authors need to explain it better and relate it with theory. It can be done in the discussion section which is quite short.
7. Data is merged inside the methodology section. Either make a different section or rename this section as “Data and Methodology”.
8. The theoretical section does not present an established theory. I would instead rename it as “Conceptual Framework”.
9. I really appreciate the depth in the analysis of this paper. However, since this tool is for policymakers, I wonder if it is feasible for the policymakers to merge so many datasets and do all these steps every time they sit for a policy meeting?

If applicable, is the statistical analysis and its interpretation appropriate?

Yes

Is the study design appropriate and is the work technically sound?

Yes

Is the work clearly and accurately presented and does it engage with the current literature?

Yes

Are the conclusions drawn adequately supported by the results?

Yes

Are sufficient details of methods and analysis provided to allow replication by others?

Partly

Are all the source data and materials underlying the results available?

Yes

Reviewer Expertise:

Economics of innovation, national system of innovation

I confirm that I have read this submission and believe that I have an appropriate level of expertise to confirm that it is of an acceptable scientific standard, however I have significant reservations, as outlined above.

# Response

**Published:** 2022-05-30

**Authors:** Levan Bzhalava

Dear Dr. Ali,

 We would like to thank you for taking the necessary time and effort to review the manuscript.

 We sincerely appreciate all your valuable comments and suggestions, which will help us to improve the quality of the manuscript.

 With best wishes,

 Levan Bzhalava

# Response

**Published:** 2022-10-12

**Authors:** Levan Bzhalava

**Affiliations:** Tallinn University of Technology, Estonia

*We would like to thank the reviewer for constructive comments and suggestions, which helped us considerably to improve the manuscript. Below we provide responses to the reviewer's comments:  *

- The authors present a novel approach to predicting signals. In order to show that their methodology is better or more sophisticated, the authors should explain how their methodology is different from the existing methodologies.

*Author Response: We included a more substantiate explanation of how the methodology proposed in the research is different from the previous ones. *

- In the methodology section, the authors write that they merge two REGPAT databases by the address of the authors. Since one of the indicators measures the impact of patents after a lag of a few years, I wonder if the addresses in the two databases are kept the same at the point of publication or if they update it later? If the latter is true, then how did the authors account for the cases where authors changed their address?

*Author Response:* *Addresses of patents are defined by OECD. We just matched OECD patent data and European Patent full text database. We made no changes or updates to addresses of patents.*

- It is not clear to me how the term “tw” is used. If it is kept fixed at 0.05 then what purpose does it serve? My guess is that the value of “tw” is conditional on the time elapsed since publication. Whatever the case might be, it needs better clarification.

*Author Response:* *Terms associated with weak signal topics usually have a low absolute occurrence frequency and a high time-weighted increasing rates. On the contrary, terms related to strong signal topics are likely to have a high absolute occurrence frequency and a high time-weighted increasing rates. In line with Yoon (2012), we study the occurrence of keywords and uses a time-weighted method (combination of time weights (tw), total number of periods (n) and specific period (j) in which term frequencies are counted) to put recent appearances of keywords more important than past appearances. For instance, in this part of  formula:* *1-tw × (n-j) , *lets calculate:  1 - 0.05 x (10-1) = 1 - 0.05 x (9)= 1 - 0.45 = 0.55 1 - 0.05x(10-2)= 1 - 0.05x(8)= 1 – 0.4 = 0.6 … 1 - 0.05x(10-10)= 1 - 0.05x(0)= 1

- I guess there are typos in Equations 1 and 2. “NNij” should be “NNj”.

*Author Response:* *Yes, there were typos in Equations 1 and 2. We corrected them. Thank you for pointing it out.*

- For those who are new to text mining, it would be helpful to know how the authors dealt with synonyms. For instance, “playing”, “lighting”, etc. can have several meanings when used as keywords. How did the authors ensure that they selected the correct patents?

​​​​​​​​​​​​​​ *Author Response:* *Previous research on text mining suggests that synonym replacement on a one-to-one word level is very likely to produce errors. We first extract only noun and adjective keywords as they represent technology concepts, and then use word2vec algorithm to group weak signal keywords. Afterward, the manual selection is used to group synonymous and to pick up main keywords associated with different sectors. After grouping weak and strong signal keywords, we use them as anchor words and utilize Correlation Explanation (CorEx) topic modelling to identify related invention activities across EU regions for the period 2010-2018.*

- How can weak signals be more likely to transform into high-impact innovations? Authors need to explain it better and relate it with theory. 

​​​​​​​​​​​​​​​​​​​​​ *Author Response: We included a more substantiate explanation of how the weak signals are more likely to transform into high-impact innovations. *

- Data is merged inside the methodology section. Either make a different section or rename this section as “Data and Methodology”.

​​​​​​​ *Author Response:* *We renamed the section as “Data and Methodology”. *

- The theoretical section does not present an established theory. I would instead rename it as “Conceptual Framework”.

​​​​​​​​​​​​​​​​​​​​​ *Author Response:* *We renamed it as “Conceptual Framework” and also* *extended literature review. *
* *

- I really appreciate the depth in the analysis of this paper. However, since this tool is for policymakers, I wonder if it is feasible for the policymakers to merge so many datasets and do all these steps every time they sit for a policy meeting?

​​​​​​​ *Author Response:* *Once data analyses are done and Python programming codes are written, most of the parts can be automated so that policymakers to have automated knowledge management tools.*
